# Unraveling the Effects of *Melissa officinalis* L. on Cognition and Sleep Quality: A Narrative Review

**DOI:** 10.3390/ijms262110566

**Published:** 2025-10-30

**Authors:** Maria Vitória Barroso Oliveira, Júlia Aparecida Garguerra, Caroline Barbalho Lamas, Lucas Fornari Laurindo, Victória Dogani Rodrigues, Kátia Portero Sloan, Lance Alan Sloan, Eduardo Federighi Baisi Chagas, Elen Landgraf Guiguer, Claudia Rucco Penteado Detregiachi, Maria Angélica Miglino, Eliana de Souza Bastos Mazuqueli Pereira, Vítor Engrácia Valenti, Luís R. Silva, Sandra Maria Barbalho

**Affiliations:** 1 Department of Biochemistry and Pharmacology, School of Medicine, Universidade de Marília (UNIMAR), Marília 17525-902, SP, Brazilefbchagas@unimar.br (E.F.B.C.);; 2 Department of Gerontology, School of Gerontology, Universidade Federal de São Carlos (UFSCar), São Carlos 13565-905, SP, Brazil; 3 Division of Cellular Growth, Hemodynamic, and Homeostasis Disorders, Graduate Program in Medical Sciences, Faculdade de Medicina, Universidade de São Paulo (USP), São Paulo 01246-903, SP, Brazil; lucasffffor@gmail.com; 4 Department of Biochemistry and Pharmacology, School of Medicine, Faculdade de Medicina de Marília (FAMEMA), Marília 17519-030, SP, Brazil; vic8dr@gmail.com; 5 Texas Institute for Kidney and Endocrine Disorders (TIKED), Lufkin, TX 75904, USA; 6 Department of Internal Medicine, University of Texas Medical Branch, Galveston, TX 77555, USA; 7 Graduate Program in Structural and Functional Interactions in Rehabilitation, School of Medicine, Universidade de Marília (UNIMAR), Marília 17525-902, SP, Brazilmiglino@unimar.br (M.A.M.);; 8 Laboratory for Systematic Investigations of Diseases, Department of Biochemistry and Pharmacology, School of Medicine, Universidade de Marília (UNIMAR), Marília 17525-902, SP, Brazil; 9 Department of Biochemistry and Nutrition, School of Food and Technology of Marília (FATEC), Marília 17500-000, SP, Brazil; 10 Postgraduate Program in Animal Health, Production and Environment, School of Veterinary Medicine, Universidade de Marília (UNIMAR), Marilia 17525-902, SP, Brazil; 11 Department of Animal Anatomy, School of Veterinary Medicine, Universidade de Marília (UNIMAR), Marília 17525-902, SP, Brazil; 12 Department of Cardiovascular and Metabolic Health, School of Philosophy and Sciences, Universidade Estadual Paulista (UNESP), Marília 17525-900, SP, Brazil; vitor.valenti@unesp.br; 13 SPRINT–Sport Physical Activity and Health Research & Innovation Center, Polytechnic of Guarda, 6300-559 Guarda, Portugal; 14 RISE-Health, Faculty of Health Sciences, University of Beira Interior, 6201-506 Covilhã, Portugal; 15 CERES, Department of Chemical Engineering, University of Coimbra, CIEPQPF, Rua Sílvio Lima, Pólo II-Pinhal de Marrocos, 3030-790 Coimbra, Portugal; 16 Department of Research, Research Coordination Center, UNIMAR Charitable Hospital, Universidade de Marília (UNIMAR), Marília 17525-902, SP, Brazil

**Keywords:** *Melissa officinalis*, Melissa, cognition, neurodegenerative diseases, sleep quality

## Abstract

The aging process is closely linked to cognitive decline, and numerous studies have demonstrated a decrease in both the quality and quantity of sleep in the general population. *Melissa officinalis* (lemon balm) is a plant rich in bioactive compounds, including flavonoids, phenolic acids, and essential oils, which are responsible for its neuroprotective and antidepressant properties. Its positive effects on the sleep quality are probably, at least in part, attributable to the presence of rosmarinic acid, which modulates *γ*-aminobutyric acid transaminase activity. This review aimed to investigate the effects of *M. officinalis* on cognition and sleep quality in human clinical trials. For cognition, studies have shown that the plant improved cognitive performance and mood. In elderly individuals with mild cognitive impairment or early Alzheimer’s disease, extracts standardized in rosmarinic acid stabilized cognitive functions and reduced neuropsychiatric symptoms such as agitation. Regarding sleep, a combination of lemon balm and valerian significantly improved sleep quality in postmenopausal women. Isolated lemon balm extracts also reduced sleep disorders in cardiac patients. When compared to citalopram, lemon balm enhanced the quality of life, including sleep quality. It is concluded that lemon balm has the potential to improve cognition and sleep quality; however, robust evidence is needed, as more rigorous trials are required.

## 1. Introduction

According to the World Health Organization (WHO), aging is a process that involves biological, psychological, and social changes, which can lead to increased vulnerability to disease and a greater need for healthcare. This process is natural and irreversible, resulting in physiological changes that affect the autonomy and quality of life of older people [1,2,3,4,5]. The increase in life expectancy leads to an irreversible global population aging. As a result, some projections indicate that by 2050, one in six people worldwide will be 65 years of age or older [5,6,7].

Modern society is facing a decline in the quality and quantity of sleep. Age and pre-existing health conditions exacerbate this situation, making older people more susceptible to sleep deprivation and dysregulation [8,9]. Sleep disorders are increasingly being seen as risk factors for dementia and accelerated cognitive impairment [10,11]. The study by Yang et al. shows that sleep quality is negatively correlated with cognitive function, with poor sleep quality potentially accelerating cognitive impairment [12,13,14]. Although changes in memory and sleep quality are often associated with the aging process, these problems are not restricted to the elderly population. Some studies show that younger adults may also experience cognitive deficits and sleep disorders, particularly in situations of intense stress or in individuals with mental health conditions, such as anxiety and depression [15,16,17,18].

Cognitive alterations, especially memory deficits, are closely related to neurodegenerative diseases. These diseases, which include Alzheimer’s disease (AD), Parkinson’s disease (PD), and others, are characterized by the progressive loss of neuronal function and structure, resulting in significant cognitive impairments, including memory loss [19,20,21].

Poor sleep quality results from a combination of factors, including stress, lifestyle habits, underlying health conditions, and environmental influences [22,23,24]. Stress, particularly among college students, has emerged as a significant factor affecting sleep, exacerbated by academic pressure and the uncertainty generated by the COVID-19 pandemic [25,26]. Habits such as excessive consumption of caffeine, alcohol, and tobacco, as well as lack of physical activity, also contribute to sleep disorders [16,27,28]. Health conditions, such as chronic respiratory diseases, psychological disorders, depression, and anxiety, have a significant impact on sleep quality [29,30,31].

Some plants have gained prominence in the medical literature due to their antioxidant effects [32,33,34,35]. *Melissa officinalis* L., commonly known as lemon balm, is often used to enhance cognitive function and improve sleep quality [36]. This medicinal plant is rich in bioactive compounds (Table 1), including flavonoids, phenolic acids, and essential oils, which are responsible for its neuroprotective and antidepressant properties, and have been studied in relation to its potential health benefits, especially in the treatment of cognitive alterations and neurodegenerative diseases, such as Alzheimer’s and Parkinson’s [37,38,39].

Some studies have shown anxiolytic, antispasmodic, antiviral, mood-modulating, and cognitive-enhancing benefits [39,63,64]. The effects on memory and cognitive function are attributed to its richness in antioxidants, including rosmarinic acid. This compound has the potential to reduce neuroinflammation and enhance mental capacity [65,66]. Its benefits on sleep quality are possibly related to the presence of rosmarinic acid, which modulates *γ*-aminobutyric acid transaminase (GABA-T) activity. This enzyme converts *γ*-aminobutyric acid (GABA) to succinate semialdehyde. GABA-T is essential for maintaining the balance of GABAergic neurotransmission, which is fundamental to brain function, including the regulation of anxiety, sleep, and epilepsy [63,67].

In view of the potential benefits of *M. officinalis* on cognition and sleep quality, this review aims to investigate its role in these areas.

## 2. Methods

### 2.1. Focused Question

This review was performed to answer the focused question: *Can M. officinalis produce beneficial effects on cognition and sleep quality?*

### 2.2. Language

Only studies in English were selected.

### 2.3. Databases

This review has included clinical trials published in PubMed (National Library of Medicine, National Institutes of Health), COCHRANE, and EMBASE. The mesh terms used were *M. officinalis* or Melissa, antioxidant, anti-inflammatory, cognition, memory, Alzheimer’s disease, Parkinson’s disease, neurodegenerative disease, sleep, or sleep quality. The use of these descriptors helped identify studies related to the plant and its health benefits on cognition and sleep quality.

### 2.4. Study Selection

Abstracts, conferences, reviews, letters to editors, and other sources were evaluated but not included. Furthermore, other relevant studies were included to help in the Introduction and Discussion sections. The inclusion criteria applied to this review were only human interventional studies. The PICO (Population, Intervention, Comparison, and Outcomes) format was used to evaluate the included clinical trials.

### 2.5. Data Extraction

We did not restrict the period of search.

## 3. *Melissa officinalis* and Conditions That Can Be Affected by This Plant

### 3.1. M. officinalis

*M. officinalis* L., popularly known as lemon balm, belongs to the *Lamiaceae* family and the *Nepetoideae* subfamily. Lemon balm can grow between 60 and 100 cm in height. Its leaves, which range from 2 to 8 cm in length, are dark green, heart-shaped, and have a rough, ribbed surface with serrated edges [64,68,69]. The plant has a hairy root system, which makes it adaptable to different environments, although its upper parts die in winter and grow back in spring; this family is widely recognized for its aromatic herbaceous plants, many of which are valued for their medicinal and nutritional properties, used in culinary and medicinal practices in various cultures. It primarily grows in the Mediterranean and Western Asia regions, but is currently cultivated in different parts of the world, including Europe, North America, and South America, such as Brazil. The plant prefers temperate climates and well-drained soils, and is frequently found in vegetable gardens, orchards, and gardens, due to its ornamental and utilitarian characteristics [37,65,70,71].

Chemical research on the composition of *M. officinalis* has revealed that the plant is rich in flavonoids, terpenoids, phenolic acids, tannins, and essential oils. These compounds can be extracted from the fresh or dried flowers, leaves, and twigs using steam distillation or chemical extraction [72,73,74]. In terms of polyphenolic compounds, the plant contains phenolic acids such as caffeic acid, caftaric acid, chlorogenic acid, ferulic acid, gentisic acid, p-coumaric acid, and rosmarinic acid, as well as flavonoids including apigenin, cynaroside, daidzein, hyperoside, isoquercetin, kaempferol, luteolin, myricetin, quercetin, quercetrol, and rutin. Volatile compounds account for (E)-caryophyllene, caryophyllene oxide, citronellal, geranial (citral A), geranyl acetate, neral (citral B), α-cadinol, α-copaene, and β-caryophyllene as major components. Triterpenes are mainly betulinic acid, oleanolic acid, and ursolic acid [37,75,76].

### 3.2. Cognition and Neurodegenerative Conditions

Human cognition involves large-scale networks that maintain functional coherence at rest and are collectively activated during cognitive processes. Cognition refers to the psychological and other processes involved in acquiring information and the capacity to understand ideas, experiences, and sensations. It is subdivided into social and non-social cognition. Non-social cognition refers to individual mental abilities, including attention, processing speed, problem-solving, reasoning skills, and working memory [77,78]. The psychological processes involved in perceiving, encoding, storing, retrieving, and regulating knowledge about oneself and others are collectively labeled as social cognition. Cognitive efficiency refers to a complex construct that represents the ability to develop learning and problem-solving skills through the optimal use of mental resources [79,80,81].

In light of this, neurodegenerative diseases are a heterogeneous group of complex conditions characterized by neuronal loss and progressive degeneration of various areas of the nervous system, impairing its functions and leading to cognitive decline [3,82,83]. These conditions, which affect the brain, retina, and spinal cord, can manifest at any stage of life. They range from congenital disorders, such as leukodystrophies, which affect the white matter during childhood, to more prevalent pathologies in aging, such as AD, PD, and age-related macular degeneration [34,84,85]. Although the exact pathogenesis of neurodegenerative diseases has not yet been fully elucidated, it is believed to result from a complex interaction between genetic, epigenetic, and environmental factors. To date, no effective therapy has been developed to slow, halt, or prevent these conditions. Therefore, understanding the molecular mechanisms underlying the pathogenesis of neurodegenerative diseases remains in high demand [82,86,87,88].

In this context, one common component among neurodegenerative diseases, such as AD, PD, and amyotrophic lateral sclerosis, is neuroinflammation [89,90,91]. In the central nervous system, microglia and astrocytes play crucial roles as regulators of inflammatory responses [92,93]. The activation of these cell types is heterogeneous and traditionally classified into two categories: neurotoxic (M1 phenotype microglia and A1 phenotype astrocytes) or neuroprotective (M2 phenotype microglia and A2 phenotype astrocytes) [94,95].

The role of the cerebral immune system is crucial in maintaining tissue homeostasis and responding to infections and injuries. Microglia, the brain’s primary resident immune cells, continuously monitor the environment and interact with astrocytes and neurons [96]. Under normal conditions, microglia remain in a state of surveillance, releasing anti-inflammatory and neuroprotective factors. However, in cases of infection or injury, they become activated, triggering an inflammatory response that eliminates pathogens and repairs damaged tissue. Ideally, this response is transient and concludes with the resolution of the pathological event [97,98]. Thereby, in situations of chronic inflammation, resolution mechanisms fail, often due to the persistent presence of inflammatory stimuli, such as misfolded protein aggregates [99,100]. These stimuli create positive feedback loops that exacerbate inflammation, leading to the production of neurotoxic factors that can worsen underlying diseases. Inflammatory responses are mediated by pattern recognition receptors, such as Toll-like receptors (TLRs), which detect foreign molecules or danger signals. For example, TLR4 recognizes bacterial lipopolysaccharides, while TLR3 detects viral RNA. These receptors can also be activated by cellular components released in response to damage, such as ATP, triggering an inflammatory cascade. The activation of these receptors initiates signaling pathways that regulate the expression of inflammatory and antimicrobial genes, such as tumor necrosis factor-*α* (TNF*α*) and interleukin (IL)-1β. These cytokines amplify the immune response and recruit other immune cells to the injury site [101,102]. While these responses are critical for defending against infections, they can cause significant collateral damage to tissues, especially in the central nervous system. In the context of neurodegenerative diseases, activated microglia release inflammatory mediators that can damage neurons, contributing to the progression of these pathologies [103,104].

### 3.3. Cognition and Inflammatory Processes

Aging brings about progressive and natural changes in cognitive function. These changes can vary from person to person, leading to different courses of aging, which can be categorized as healthy aging (considered normal) or pathological [105,106,107]. Neuroinflammation, which plays a role in correcting and resolving neural tissue damage, is an immune response to infections that can eliminate pathogens and cellular debris from the central nervous system. A priori, they are beneficial to neural tissue; however, in chronic neurological diseases, they become persistent and harmful to neurons [108,109,110].

Neurodegenerative diseases such as AD, amyotrophic lateral sclerosis, and PD have in common neuroinflammation mediated by microglia, which can assume an M1 or M2 phenotype. Depending on which phenotype is activated, these cells may have a neuroprotective or cytotoxic effect. Microglia with the M1 phenotype are associated with the production of pro-inflammatory cytokines, such as TNF-*α*, IL-1*β*, and IL-6, IL-18, and IL-12, reactive oxygen species (ROS), nitric oxide, glutamate, and prostaglandins, as well as enzymes such as matrix metalloproteinases (MMPs). In contrast, the M2 phenotype is associated with anti-inflammatory effects [110,111,112].

The accumulation of proteins and the dysregulation of mechanisms controlling inflammation are among the various causes of inflammation in neurodegenerative disorders. The primary one is the aggregation of proteins resulting from genetic mutations in specific proteins. For example, it is worth mentioning the mutations in the genes of the human amyloid precursor protein that cause early-onset AD, as well as the mutations in proteins that encode the ubiquitin E3 ligase parkin, which can cause familial PD [113,114,115].

Currently, studies describe the mechanisms associated with neuroinflammation due to the accumulation of misfolded proteins in neurodegenerative diseases [116,117]. The interaction that occurs between microglia, neurons and astrocytes, in the nervous system, during neurodegenerative diseases leads to the activation of the nuclear factor-*κ*B (NF-*κ*B) pathway, which, when translocated to the cell nucleus, promotes the transcription of several cytokines and pro-inflammatory chemokines, including pyrin-containing inflammasome 3 (NLRP3), triggering a cascade of inflammation that leads to behavioral deficits and neuronal death [118,119].

**Figure 1:**
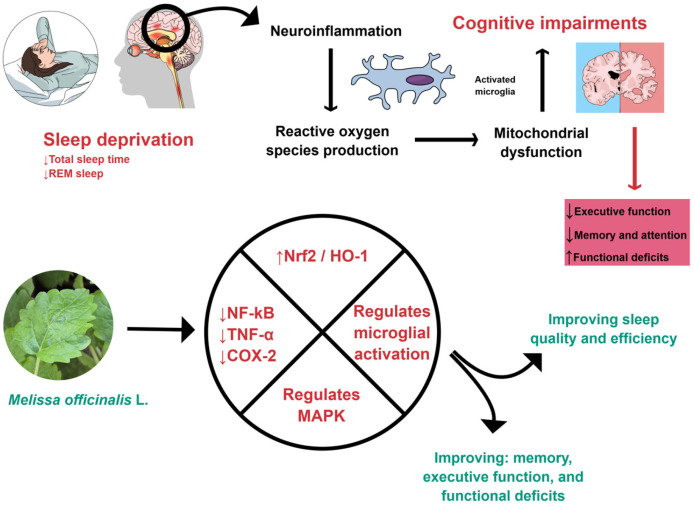
*M. officinalis* can interfere with inflammatory processes by down-regulating NF-kB, TNF-*α*, and COX-2. Furthermore, it can promote an increase in Nrf2 and HO-1, regulate microglial activation, reduce neuroinflammation, and improve sleep. COX-2: Cyclooxygenase-2; HO-1, Heme-oxygenase 1; MAPK, Mitogen-activated protein kinase; NF-kB: Nuclear Factor Kappa Beta; Nrf2: Nuclear factor erythroid 2-related factor 2; REM, Rapid eye movement; TNF-α: Tumor Necrosis Factor-alpha. Created using Mind the Graph (https://mindthegraph.com/), accessed on 17 October 2025.

**Figure 2:**
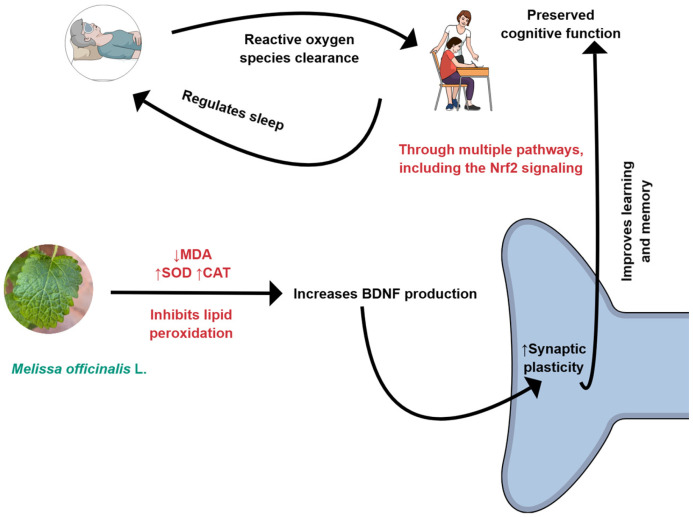
*M. officinalis* can reduce oxidative stress by decreasing the production of MDA (malondialdehyde) and increasing the levels of CAT (catalase) and SOD (superoxide dismutase). BDNF, Brain-derived neurotrophic factor; Nrf2: Nuclear factor erythroid 2-related factor 2. Created using Mind the Graph (https://mindthegraph.com/), accessed on 17 October 2025.

*M. officinalis* can affect oxidative and inflammatory processes, as shown in Figure 1 and Figure 2. For these reasons, it can improve cognition.

### 3.4. Cognition and Oxidative Stress

Oxidative stress is characterized by an imbalance between the production of ROS and antioxidant substances, which are responsible for protecting the body from free radicals, leading to oxidative damage to cells [120,121]. The brain is susceptible to neurodegeneration, which is the progressive and irreversible loss of interference in the nervous system due to damage to neurons. This occurs when the brain is exposed to an accumulation of ROS, which have lipid components capable of promoting a cascade of redox reactions, which cause damage to cells and compromise their functions [122,123,124].

Neuronal injuries caused by oxidative stress, which result in the interruption of synaptic transmission and neuronal dysfunction due to changes in DNA, proteins, and lipids, generally occur in more susceptible areas, such as the hippocampus and regions responsible for memory storage [125,126]. Cognitive deficits resulting from neuronal injuries can be classified according to their impact on the person’s quality of life, which can be disabling for both the patient and those around them [127].

### 3.5. Sleep Quality

Sleep is a physiological component of the human being, with a strong influence on cognitive processing [128]. However, as age progresses, older adults tend to experience more sleep disorders than young people [129,130]. Sleep pattern disorders are considered health problems that interfere with a person’s ability to sleep consistently and without interruptions for an extended period, and can be associated with pathologies, such as depressive disorders, anxiety, post-traumatic events, as well as stressful episodes of everyday life. The main manifestations of sleep disorders are insomnia, sleep apnea, and restlessness [131,132].

Sleep disorders are associated with numerous pathologies, including neurodegenerative diseases, which have a bidirectional association with changes in sleep patterns [133,134]. There are mechanisms by which sleep disorders and the risks of neurodegeneration are associated, among them it is worth mentioning, the production and aggregation of *β*-amyloid (A*β*), due to the increase in synaptic neuronal activity, which is naturally removed during sleep and when there is a change in the sleep pattern, this removal does not occur, leading to the accumulation of this substance and the activation of the inflammatory process with secretion of pro-inflammatory cytokines, due to the stimulation of inflammatory pathways when there is a change in the sleep pattern. Furthermore, the presence of pro-inflammatory cytokines, such as IL-6 and TNF-*α*, results in greater stimulation of A*β* production and aggregation, which in turn stimulates the secretion of more inflammatory cytokines and amplifies neurodegeneration [135,136].

In general, sleep disorders affect the functioning of the human body, leading to changes such as a decreased pain threshold, which increases sensitivity and perception of pain stimuli. Moreover, it is related to anxiety, reduction in memory, and neurodegenerative diseases. During sleep, the body produces endorphins and other neurochemical substances that can help control pain [137,138]. *M. officinalis* can interfere in oxidative and inflammatory processes, as shown in Figure 1 and Figure 2. For these reasons, it can improve sleep quality.

## 4. Clinical Trials That Evaluated the Effects of *M. officinalis* on Cognition

As mentioned above, *M. officinalis* is known to influence cognition and sleep quality. The effects on cognition are shown in Table 2. This table presents a variety of clinical studies that investigated the impact of *M. officinalis* on cognitive function. These studies vary in design, population, dosage, and outcome measures, but collectively provide insight into the potential mental effects of *M. officinalis* in both healthy individuals and those with cognitive impairments. Although this review provides an overview of the current evidence regarding the effects of *M. officinalis* L. on cognition and sleep quality, it is essential to recognize that most of the available clinical trials are small, single-center studies with limited sample sizes, which may restrict the generalizability of their findings. Furthermore, the predominance of studies reporting positive results raises the possibility of publication bias, as trials with null or adverse outcomes may be underrepresented in the literature. Future research should therefore include larger, multicenter randomized controlled trials with standardized methodologies to confirm and extend these preliminary findings.

The randomized, double-blind, placebo-controlled, crossover trial conducted by Kennedy et al. investigated the acute effects of *M. officinalis* (lemon balm) extract on cognition and mood in 20 healthy young volunteers [139]. Participants received single doses of 600 mg, 1000 mg, or 1600 mg of *M. officinalis* (encapsulated dried leaf) or a placebo. Cognitive performance and mood were assessed before and after administration. Results demonstrated significant improvements in cognitive task performance and mood, particularly with the 1600 mg dose. No adverse effects were reported. Although this is a critical study, the sample size is too restricted. Furthermore, the study evaluated only the single-dose acute impact, which does not provide information on chronic use effects and long-term safety. These aspects interfere with the conclusions of this study.

The randomized, placebo-controlled trial by Watson et al. investigated the impact of aromatherapy using lavender (*Lavandula angustifolia*) and lemon balm (*M. officinalis*) essential oils on agitation in older adults, with and without dementia [140]. Participants received once-daily treatments with lavender, lemon balm, or placebo oil (sunflower oil) for 2 weeks. The study found no statistically significant differences in agitation levels between the treatment groups and the placebo group. No significant adverse effects were reported.

Noguchi-Shinohara et al. conducted a study to evaluate the effects of rosmarinic acid, present in *M. officinalis* extract, in patients with dementia associated with AD [141]. A total of 23 patients participated in this study, who were randomized into two groups (*M. officinalis* extract and placebo groups). During 24 weeks, participants received daily doses of either 500 mg of rosmarinic acid in *M. officinalis* extract or a placebo. The study lasted an additional 24 weeks (washout period), totaling 48 weeks of evaluation, during which all participants were assigned to the *M. officinalis* extract group. The results suggested that the treatment can help in the prevention of neuropsychiatric symptoms related to AD. Despite the promising results, the study has limitations, including a small sample size, which necessitates a more cautious interpretation of the findings. Moreover, this study may have other limitations, such as the inclusion of heterogeneous patients.

Noguchi-Shinohara et al. conducted a study aiming to evaluate the effects of *M. officinalis* on cognition in older adults [142]. For this, 323 participants with subjective or mild cognitive impairment were randomized into two groups: the *M. officinalis* group and the placebo group. The study had two phases. Phase 1, which corresponds to the intervention lasting 96 weeks, and phase 2, which was a washout period of 24 weeks. The results obtained indicate that *M. officinalis* extract can help prevent cognitive decline in older adults without hypertension. The randomized, double-masked, placebo-controlled study design is its strong point. However, the sample size and the failure to analyze variables such as amyloid and tau protein limited the study’s findings.

The studies included in this narrative review exhibit notable methodological limitations and considerable heterogeneity, which warrant careful interpretation of their findings. First, many of the trials had relatively small sample sizes, which limits the statistical power and generalizability of their results. In some cases, convenience sampling and inadequate randomization procedures further undermine the reliability of the reported outcomes.

Additionally, there was significant variation in study designs, intervention protocols, and outcome measures across the trials. The dosage and duration of *M. officinalis* administration varied widely, ranging from single acute doses to chronic administration over several weeks, and included both standardized extracts and combinations with other herbal compounds (e.g., valerian). This heterogeneity complicates direct comparisons between studies and precludes the possibility of performing a meta-analysis.

Moreover, most studies relied on subjective assessment tools, such as self-reported questionnaires, to evaluate cognitive performance and sleep quality, which are susceptible to bias. Objective measures, including neuroimaging biomarkers or polysomnography, were rarely employed. The lack of standardized and validated outcome measures contributes to the difficulty in synthesizing results across studies.

Blinding and allocation concealment procedures were insufficiently described in several trials, introducing a potential risk of bias. Furthermore, only a few studies reported monitoring or controlling for confounding variables such as concomitant medication use, lifestyle factors, or comorbid conditions, all of which could have influenced the outcomes.

When comparing trials with positive outcomes to those reporting null results, several methodological and population-related differences emerge. For instance, Kennedy et al. [139] reported acute improvements in cognitive performance and mood following single doses of standardized *M. officinalis* extract in healthy young adults. In contrast, Noguchi-Shinohara et al. [142] observed no significant cognitive improvements after long-term supplementation in older adults with mild cognitive impairment. These discrepancies may be attributed to differences in the study populations (healthy versus cognitively impaired), duration of intervention (acute versus chronic), and the specific formulations used (standardized extract with defined rosmarinic acid content versus a more variable preparation). Similarly, while aromatherapy trials using *M. officinalis* essential oil [140] yielded null results, these interventions employed administration routes other than oral administration, which may have limited bioavailability and central nervous system effects. Overall, the variability in study design, extract composition, and participant characteristics seems to partly explain the inconsistent findings across clinical trials evaluating *M. officinalis* and cognition.

## 5. Clinical Trials That Evaluated the Effects of *M. officinalis* on Sleep Quality

The effects of *M. officinalis* on sleep quality are shown in Table 3. This table showcases a range of clinical studies examining the effects of *M. officinalis* on sleep quality. As the previous table investigated the effects of *M. officinalis* on cognitive function, Table 3 also presents studies that differ in their design, population, dosage, and outcome measures. Together, these selected studies shed light on the potential mental benefits of *M. officinalis* for individuals with sleep disturbances.

In a clinical trial involving 13 men and 17 women, the authors used a highly standardized *M. officinalis* extract, formulated as Phytosome (MOP), and observed a significant improvement in sleep quality [63]. The results of this study are critical because they suggest a beneficial effect of MOP on subjective sleep quality in adults with mild to moderate insomnia. However, this study has limitations, including a small sample size and the need to evaluate different doses and long-term outcomes to confirm these findings.

An interesting study evaluated the effects of *M. officinalis* combined with valerian on menopausal women [143]. Their results showed significant effects on sleep quality. Nevertheless, some limitations are related to this trial, such as the combination of the plants.

The study conducted by Haybar et al. evaluated the effects of *M. officinalis* in patients with angina and showed significant amelioration of anxiety, depression, and sleep disorders [144].

Another clinical trial demonstrated that the use of *M. officinalis* could improve all MENQOL domain scores compared to the citalopram or placebo groups; consequently, it is possible to infer that there was an improvement in sleep quality. However, as a limitation, the study presented a small sample size and a limited intervention duration. Furthermore, lifestyle factors and medications were not controlled [145].

Some limitations can be reported in the studies related to *M. officinalis* effects both in cognition and sleep quality; the benefits found are more consistent in specific populations (such as menopause and angina), but are frequently associated with other herbs such as valerian. In addition, the samples are usually small, there are issues with blinding in some studies, and the trials employ subjective outcome measures.

A notable limitation of the current body of evidence on *M. officinalis* is the potential for publication bias. Many of the included studies are small-scale trials reporting positive outcomes, which raises concerns about the underreporting of negative or inconclusive findings. This bias may overestimate the perceived efficacy of *M. officinalis* in improving cognition and sleep quality, as studies with unfavorable results are less likely to be published or indexed in major databases. A systematic effort to include unpublished data and gray literature would be essential to mitigate this risk in future reviews.

Furthermore, there is a marked lack of standardization in the dosing regimens of *M. officinalis* used across studies. Dosages varied widely, with differences in the form of administration (e.g., capsules, extracts, or combined formulations with other herbs such as valerian). These inconsistencies make it difficult to identify an optimal therapeutic dose or to compare results across trials. The absence of standardized extracts and uniform reporting of bioactive compound concentrations, such as rosmarinic acid levels, further limits the ability to draw robust conclusions.

To strengthen the evidence base, future research should prioritize the use of standardized *M. officinalis* preparations with well-defined concentrations of active constituents. Dose-response studies and trials using harmonized protocols are needed to determine effective and safe dosing strategies.

While several trials examining the effects of *M. officinalis* on sleep quality report positive results, there is insufficient critical comparison of these findings with those from studies reporting null results. For example, in the trial by Di Pierro et al. [63], significant improvements in sleep quality were observed in the treatment group, with 87% of participants experiencing an improvement in sleep quality compared to just 30% in the placebo group. In contrast, the trial by Shirazi et al. [145] showed that while *M. officinalis* was more effective than placebo and citalopram in improving various quality-of-life scores in postmenopausal women, the study did not provide a robust comparison of the magnitude of effects across different interventions. Additionally, the trial by Taavoni et al. [143] reported improvements in sleep quality in menopausal women, but without clearly contrasting the effects with studies that failed to show such benefits. A more balanced presentation of both positive and null findings would help contextualize the potential efficacy of *M. officinalis* in sleep quality improvement and give readers a clearer understanding of the treatment’s effectiveness relative to other interventions.

Although preclinical studies provide robust evidence that *M. officinalis* can modulate key neurobiological pathways—such as reducing oxidative stress and downregulating pro-inflammatory mediators—these mechanistic effects have not yet been conclusively demonstrated in human populations. Small sample sizes, short durations, heterogeneous populations, and variability in the form and standardization of *M. officinalis* extracts limit most clinical trials conducted to date. Moreover, most trials rely on subjective outcome measures (e.g., self-reported sleep quality or cognitive assessments) and lack objective biomarkers that could confirm engagement of the proposed molecular targets. No studies to date have measured inflammatory or oxidative stress markers (such as cytokine levels, Nrf2 activation, or oxidative stress biomarkers) in clinical contexts, limiting the ability to establish a transparent mechanistic bridge.

Therefore, while the biological plausibility of *M. officinalis* as a neuroprotective agent is supported by mechanistic studies in vitro and in vivo, the current clinical evidence remains preliminary. To strengthen the translational link, future trials should incorporate standardized extracts with defined concentrations of bioactive compounds (e.g., rosmarinic acid), utilize objective and validated outcome measures, and assess biological endpoints that align with preclinical mechanisms. This approach would allow for a more rigorous evaluation of the therapeutic potential of *M. officinalis* in cognitive and sleep-related disorders.

## 6. Conclusions and Future Perspectives

The available evidence suggests that *M. officinalis* L. may exert beneficial effects on specific aspects of cognition and sleep quality. This supports its use as a nutraceutical supplement to ameliorate people’s well-being regarding sleep disorders and cognition-related deficits, including those related to anxiety and stress. However, caution is needed due to the included studies’ heterogeneity and methodological limitations.

*M. officinalis* L.’s bioactive compounds, such as flavonoids and phenolic acids, appear to have a positive influence on brain function and neurodegenerative processes. Studies suggest that lemon balm extract can enhance cognitive performance and mood, particularly at moderate doses, with minimal adverse effects. Additionally, the evidence points to its potential in alleviating neuropsychiatric symptoms associated with neurodegenerative diseases like AD, which may help prevent cognitive decline in older adults without dementia. However, while the results are promising, there are still limitations, such as small sample sizes in some studies and a lack of deeper analyses of biomarkers like amyloid and tau, which are crucial for understanding the underlying mechanisms of the plant’s effects on the brain. Further clinical studies with larger sample sizes and more extended follow-up periods are necessary to confirm these findings and explore the therapeutic potential of *M. officinalis*. Future studies should also prioritize robust cohorts to enhance the statistical reliability and generalizability of their results. In addition to increasing sample size, it is necessary to investigate the effects in groups of different ethnicities, elderly individuals, and those with comorbidities, and explore the effects in younger populations with chronic stress or anxiety.

Other limitations of the current literature involve a lack of long-term/chronic studies, which could raise concerns about safety and sustained efficacy. Additionally, the included studies often comprised heterogeneous designs and dosing regimens, which limit the identification of optimal doses or the comparison of results with the utmost clarity. Furthermore, some studies combined *M. officinalis* with other herbs, such as valerian, which makes it unclear whether the effects studied are due to *M. officinalis* itself.

In this sense, larger and adequately powered randomized clinical studies should be tailored to enhance the generalizability of findings and to confirm efficacy across diverse populations. The cohorts should also be studied under more extended intervention periods to assess sustained effects and safety over chronic use. Standardization of *M. officinalis* preparations should also be prioritized, including the quantification of key bioactive compounds such as rosmarinic acid, which is crucial to determine dose-response relationships and enable cross-trial comparisons.

Rigorous randomization and blinding procedures are necessary in this regard, along with careful monitoring of potential cofounders such as concomitant medications, lifestyle factors, and comorbid conditions. To overcome the limitations of subjective assessments, future studies should prioritize other methods or objective biomarkers to assess treatment results. These include neuroimaging and electrophysiological measures for cognition, as well as polysomnography or actigraphy for sleep quality.

**Figure 3:**
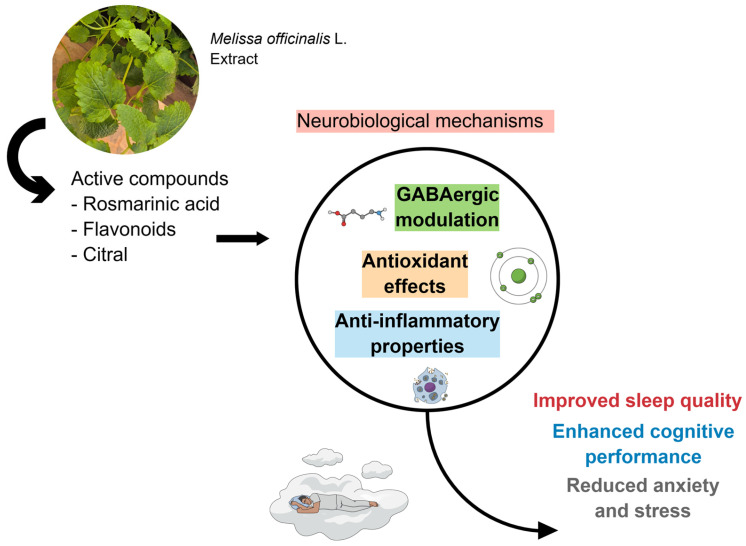
Due to its bioactive compounds, *M. officinalis* can effectively trigger GABAergic modulation, promote antioxidant effects, and exert anti-inflammatory properties. These actions modulate sleep quality, enhance cognitive performance, and reduce anxiety and stress. Created using Mind the Graph (https://mindthegraph.com/), accessed on 17 October 2025.

The evaluation of inflammatory and oxidative stress markers, as well as cognitive markers, can significantly help in understanding the effects produced by the plant. Standardization of the strata used, along with objective measures for assessing sleep and cognition, would provide more in-depth explanations of the effects of *M. officinalis*. Figure 3 shows the results of the included clinical studies, considering the biological effects attributed to *M. officinalis*.

## Figures and Tables

**Table 1:** Some bioactive compounds are found in *M. officinalis*.

Bioactive Compound	Structure	Functions	Reference
Volatile Compounds			
Geranial (Citral A)	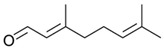	Antifungal, antibacterial	[37,40]
Neral (Citral B)	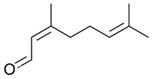	Antifungal, antibacterial	[37,41,42]
Citronellal	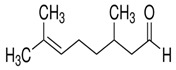	Antimicrobial, insecticidal	[37,43,44,45]
Triterpenes			
Ursolic acid	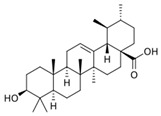	Antibacterial, antioxidant	[37,46,47,48]
Oleanolic acid	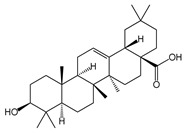	Antiviral, hepatoprotective, antioxidant	[37,49,50]
Phenolic Compounds			
Rosmarinic acid	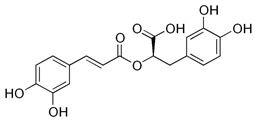	Antioxidant, anti-inflammatory	[37,51,52,53,54]
Caffeic acid	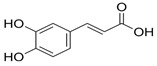	Antioxidant, anti-inflammatory	[37,55,56]
Flavonoids			
Quercetin	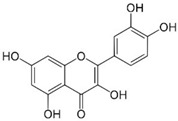	Anti-inflammatory, antioxidant	[37,57,58,59]
Luteolin	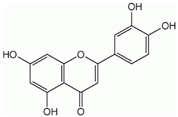	Antioxidant, anti-inflammatory, immune system modulator, fatty acid synthase inhibitor	[60,61,62]

**Table 2:** Clinical trials that evaluated the effects of *M. officinalis* on cognition.

Model/Country	Population	Intervention/Comparison	Outcomes	Side Effects	Reference
Randomized, double-blind, placebo-controlled, crossover study/United Kingdom	20 healthy young volunteers.	Participants received acute single doses of 600 mg, 1000 mg, and 1600 mg of encapsulated dried leaf *M. officinalis*, compared to a placebo.	Improvements in cognitive performance and positive mood effects were observed, particularly with the dose of 1600 mg of *M. officinalis*.	NR by patients	[139]
Randomized, placebo-controlled trial/Australia	49 older adults, with and without dementia, exhibiting agitated behaviors.	Participants were randomized to receive aromatherapy treatment with either lavender (*Lavandula angustifolia*) essential oil, lemon balm (*M. officinalis*) essential oil, or a placebo (sunflower oil) once daily for 2 weeks.	The study found no statistically significant differences in agitation levels between the treatment groups and the placebo group, independent of cognitive groups.	NR by patients	[140]
Randomized, double-blind, placebo-controlled, 24-week trial, with an open-label extension to 48 weeks/Japan	23 patients (12 were in the *M. officinalis* extract group and 11 in the placebo group) with mild dementia, due to AD.	Patients were randomly assigned in a 1:1 ratio to receive the intervention (*M. officinalis* extract containing 500 mg of RA daily) or placebo, based on age and sex, for 24 weeks. In the extension period (an additional 24 weeks), all participants were allocated to the *M. officinalis* extract group.	The results suggested that RA may help prevent the worsening of AD-related neuropsychiatric symptoms.	The side effects reported during the extension period were bruising, headache, and hematuria.	[141]
Randomized, placebo-controlled, double-blind study/Japan	323 individuals diagnosed with subjective or mild cognitive impairment aged between 65 and 79 years.	In phase 1, participants were randomized into *M. officinalis* group (*n* = 162, 185 mg of *M. officinalis* extract along with 50 mg of RA, 60 mg of lactose and 5 mg of calcium stearate) and placebo (*n* = 161, 210 mg of lactose, 35 mg of caramel and 5 mg of calcium stearate) for 96 weeks. Phase 2 was a washout period of 24 weeks for all individuals.	The results showed no significant differences in cognitive measures between the placebo and *M. officinalis* groups from baseline to 96 weeks. However, based on the analysis of CDR-SB scores in participants without hypertension, the score increased by 0.006 and decreased by 0.085 in the *M. officinalis* and placebo groups, respectively (*p* = 0.036).	116 participants in the *M. officinalis* group and 114 in the placebo group reported adverse effects such as trauma, skin disease, orthopedic, cardiovascular disease, etc.	[142]

AD: Alzheimer’s disease; RA: rosmarinic acid; CDR-SB: Clinical Dementia Rating Sum of Boxes; NR, not reported.

**Table 3:** Clinical trials showing the effects of *M. officinalis* on sleep quality.

Reference	Model/Country	Population	Intervention/Comparison	Outcomes	Side Effects
[63]	Prospective, double-blind, placebo-controlled, and crossover study/Italy	30 individuals (13 men and 17 women); 18–65 years, and perception of fatigue upon waking and unrefreshing sleep.	Patients received two tablets of *M. officinalis* highly standardized extract, formulated as Phytosome (MOP) or a placebo, every evening, 30 min before bedtime.	There was a significant reduction in the ISI score in the treated group, with 87% of the treated group having improvement in sleep quality, compared to 30% in the placebo group.	NR by patients
[143]	Randomized, triple-blind, placebo-controlled trial/Iran	100 menopausal women aged 50–60 years experiencing sleep disturbances.	Intervention group (*n* = 50) received two capsules daily containing 160 mg of essence of *Valeriana officinalis* and 80 mg of *Melissa officinalis* (lemon balm). Placebo group (*n* = 50) received capsules containing 50 mg starch.	Significant improvement in sleep quality was observed in the treatment group, as measured by the PSQI.	NR by patients
[144]	Randomized, double-blind, placebo-controlled trial/Iran	80 patients aged 40–75 years with chronic stable angina.	Participants received 3 g per day of *M. officinalis* (*n* = 40) or a placebo (*n* = 40) for 8 weeks.	Significant reductions in depression, anxiety, stress, and sleep disorders were observed in the *M. officinalis* group.	NR by patients
[145]	Randomized, double-blind clinical trial/Iran	60 postmenopausal women (average age: 51.9 years) with menopause confirmation tests (FSH > 40 mIU/mL, and estradiol < 20 pg/mL), who slept poorly according to the PSQI.	Participants were randomized into three groups of 20 people, with one group receiving 30 mg of citalopram, another receiving one capsule of 500 mg *M. officinalis*, and the third group a 500 mg capsule of starch (placebo) once daily for 8 weeks.	All MENQOL domain scores improved significantly in the *M. officinalis* group compared to citalopram and placebo (*p* < 0.001).	Two participants in the citalopram group reported nausea, and one reported headache; no reports of adverse effects in the *M. officinalis* and placebo groups.

FSH, Follicle-stimulating hormone; ISI: Insomnia Severity Index; MENQOL: Menopause-Specific Quality of Life questionnaire; NR, Not reported; PSQI: Pittsburgh Sleep Quality Index.

## Data Availability

No new data were created or analyzed in this study. Data sharing is not applicable to this article.
